# 787. Clinical and Genomic Epidemiology of *mcr-9* Containing Carbapenem-resistant *Enterobacterales* Isolates in Metropolitan Atlanta, 2012-2017

**DOI:** 10.1093/ofid/ofab466.984

**Published:** 2021-12-04

**Authors:** Ahmed Babiker, Chris W Bower, Sarah W Satola, Jesse T Jacob, Michael H Woodworth

**Affiliations:** 1 Emory University School of Medicine, Atlanta, GA; 2 Georgia Emerging Infections Program, Decatur, GA; 3 Emory University, Atlanta, GA

## Abstract

**Background:**

Colistin is a last-resort antibiotic for multidrug resistant gram-negative infections. Recently, a new allele of the mobile colistin resistance (*mcr*) gene family designated *mcr-9,* has been reported. However, its clinical and phenotypic significance remains unclear.

**Methods:**

The Centers for Diseases Control and Prevention-funded Georgia Emerging Infections Program (EIP) performs population- and laboratory- based surveillance for CRE isolated from sterile sites or urine in metropolitan Atlanta, GA including standardized chart abstraction. We queried genomes of carbapenem-resistant Enterobacterales (CRE) for *mcr-9* from a convenience sample of Georgia EIP clinical isolates between 2012-2017. Isolates underwent phenotypic characterization by broth microdilution and population analysis profiling. Nine available *E. cloacae* (two *mcr-9* positive, seven *mcr-9* negative) genomes from the National Institutes of Health were included in downstream genomic analysis. Fastq files underwent *de novo* assembly, annotation and AMR and virulence gene prediction, pan-genome association analysis, pairwise comparisons of average nucleotide identity and phylogenetic tree construction based on core genes. We compared characteristics and outcomes of *mcr-9* positive and negative CRE cases.

**Results:**

Among 449 sequenced CRE genomes, thirteen (2.9%) were found to harbor *mcr-9*, all of which were *E. cloacae.* Fourteen *mcr-9* negative E*. cloacae* (n=14) were included as a comparative group. *E. cloacae* was most commonly isolated from the urine (22/24, 86%), and none were community associated. The median colistin MIC, rates of heteroresistance and inducible resistance were similar between *mcr-9* positive and negative isolates (Table 1). 90-day mortality was high in both *mcr-9* positive (31%) and negative (7% cases (p=0.28, Table 1). Phylogenetic analysis revealed no geo-temporal clustering (Figure 1). Plasmid-associated genes were significantly associated with the presence of *mcr-9* (p< 0.001). Phylogeny and average nucleotide identity heatmap of mcr-9 positive and mcr-9 negative E. cloacae.

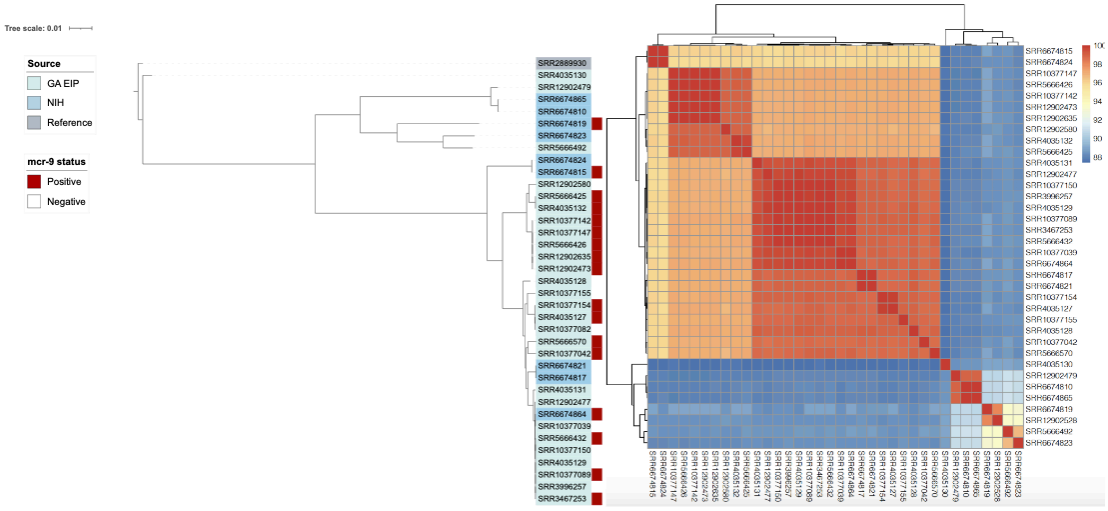

Figure Legend 1: Phylogeny and average nucleotide identity heatmap of mcr-9 positive (n=13) and mcr-9 negative (n=14) E. cloacae from Georgia Emerging Infection program in addition to 9 available E. cloacae (two mcr-9 positive, seven mcr-9 negative) from the National Institutes of Health. A phylogenetic tree based on a core gene alignment containing 1,904 genes defined using Roary v3.13.0. was generated using IQtree v2.0.3. A maximum likelihood tree was generated by running 1,000 bootstrap replicates under the generalized time-reversible model of evolution. The tree was visualized and annotated using Interactive Tree of Life (iTOL) v4. Pairwise comparisons of average nucleotide identity on the assembled genomes were performed with the Mashmap method using fastANI v1.32. Abbreviations: GA EIP: Georgia Emerging Infection Program, NIH: National Institutes of Health,

Table 1: Carbapenem-resistant E. cloacae clinical and microbiological characteristics

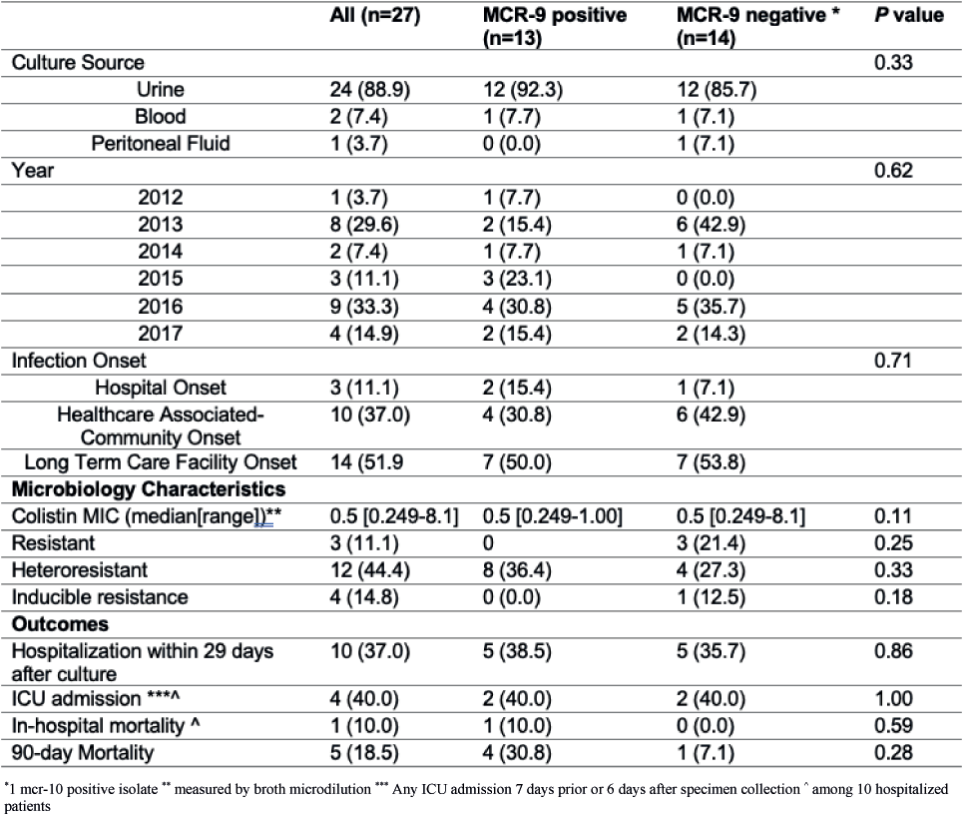

**Conclusion:**

The presence of *mcr-9* was not associated with significant changes in colistin resistance or clinical outcomes.

**Disclosures:**

**All Authors**: No reported disclosures

